# Validation of the Arabic version of the modified Yale Food Addiction Scale in the general population in Lebanon

**DOI:** 10.1186/s40337-022-00638-2

**Published:** 2022-08-04

**Authors:** Souheil Hallit, Anna Brytek-Matera, Diana Malaeb, Sahar Obeid

**Affiliations:** 1 School of Medicine and Medical Sciences, Holy Spirit University of Kaslik, P.O. Box 446, Jounieh, Lebanon; 2 Psychology Department, College of Humanities, Effat University, Jeddah, 21478 Saudi Arabia; 3 Research Department, Psychiatric Hospital of the Cross, Jal Eddib, Lebanon; 4 Institute of Psychology, University of Wroclaw, Dawida 1, 50-527 Wrocław, Poland; 5 College of Pharmacy, Gulf Medical University, Ajman, United Arab Emirates; 6 School of Pharmacy, Lebanese International University, Beirut, Lebanon; 7 School of Arts and Sciences, Social and Education Sciences Department, Lebanese American University, Jbeil, Lebanon

**Keywords:** mYFAS, Food addiction, Validation, Psychometric properties, Arabic, Lebanon

## Abstract

**Background:**

Although the definition of food addiction is not agreed upon, it is characterized by eating more than expected without being hungry, not being able to visit certain places associated with overeating or unsuccessfully trying to cut down on the consumption of certain foods. The modified YFAS (mYFAS 2.0) version, instrument available to evaluate food addiction, was shown to have good psychometric properties. Our objective was to assess the psychometric properties of the Arabic version of the mYFAS (mYFAS-Ar-Leb) in the Lebanese population.

**Methods:**

This cross-sectional study enrolled 1268 persons residing in Lebanon (September–November 2020). The mean age of the participants was 26.18 years (SD = 11.17; min: 18; max: 85), with 65.1% females. The percentage of participants with food addiction was 226 (17.8%) in the total sample. A confirmatory factor analysis was run on the one-factor structure among the total sample.

**Results:**

The fit indices of the confirmatory factor analysis of the scale were excellent. The Cronbach’s alpha value was good for the total scale. The mYFAS-Ar-Leb score was positively and strongly associated with stress, anxiety and depression.

**Conclusion:**

Our study findings highlighted that the use of the mYFAS-Ar-Leb in Lebanese population might help estimate food addiction prevalence and stress on the need for effective treatment and preventive measures to craving for addictive foods.

## Plain English Summary

Food addiction is characterized by eating more than expected without being hungry, not being able to visit certain places associated with overeating or unsuccessfully trying to cut down on the consumption of certain foods. The modified YFAS (mYFAS 2.0) version is a tool used to assess food addiction. Our objective was to assess the psychometric properties of the Arabic version of the mYFAS (mYFAS-Ar-Leb) in the Lebanese population. The results we obtained were excellent in terms of confirmatory factor analysis, reliability and divergent validity. They highlighted that the mYFAS-Ar-Leb might be used for the estimation of food addiction among the Lebanese population.

## Background

Obesity, a major health risk, is on the rise worldwide [1], with the sustainability of weight loss with contemporary treatments remaining a challenge. Obesity has been described as a global epidemic with 36.9% males and 38.0% females worldwide classified as overweight or obese [2]. In countries of the Gulf region, there is a significant increase in obesity with a prevalence of 2–55% in adult females and 1–30% in adult males [3]. In Lebanon, a cross-sectional survey conducted in 1997 and 2009 showed a tremendous growth in the prevalence of overweight and obesity over a 12-year period (17.4% vs. 28.2% in adults) [4]. Another study suggested that the prevalence of obese subjects (BMI > 30 kg/m^2^) was 18.16%, with the percentage of males being more than double that of females (24.86% vs 10.82%) [5].

Some people struggle to regulate their food intake [6]. In some cases, food addiction is manifested by the loss of control over particular foods that are high in sugar, fat, and salt [7, 8]. These foods are similar to addictive drugs and alcohol since they can interfere with the reward mechanism of the brain’s limbic system [9]. Hence, the term "food addiction", proposed by Randolph in 1956, emphasizes the excessive/compulsive consumption of these specific foods [10]. Food addiction shares many common features with substance abuse, but is not considered a disorder in the Diagnostic and Statistical Manual of Mental Disorders (DSM-5) yet; the integration of food addiction into the substance use disorders umbrella requires a chemical to be associated with addiction for which there is insufficient evidence to consider a defined chemical in everyday food that can unequivocally induce addiction [11, 12]. Food addiction includes craving for foods that are high in sugar or fat without the investigation of the failure to fulfill a major obligation at work or school [12]. This raises the debate about whether food addiction is a new concept of problematic eating behaviors or not [10].

Food addiction is defined as “hedonic eating behavior involving the consumption of highly palatable foods (i.e. foods high in salt, fat, and sugar) in quantities beyond homeostatic energy requirements” [13]. Previous studies revealed a strong overlap between symptoms of food addiction and binge eating [14, 15], while others pointed out that there are specific physiological correlates of addictive eating that make this construct more similar to a substance use disorder subtype [10, 16]. In fact, mental health issues were shown to increase food addiction according to a meta-analysis [17]. In addition, food addiction is influenced by gender, type of food content especially high in sweets, and genetics [18–20].

Although overweight and obesity denote the most apparent consequences of food addiction, only some people with obesity display signs of food addiction [21]. Previous research showed that the food addiction prevalence among binge eaters was 56.8% [17], with 25% of individuals with obesity seeking weight loss treatment [22]; therefore, it was concluded that these two concepts are closely related, and might overlap [23].

Both the Yale Food Addiction Scale (YFAS) and modified YFAS (mYFAS 2.0) are measuring instruments available to assess food addiction [24, 25]. The YFAS was initially constructed in 2009 based on the diagnostic criteria for substance use disorders of the DSM-4 Text Revision [26, 27]. The YFAS is a self-report measure, consisting of 25 items assessing addiction to highly palatable foods (such as chocolate, ice cream and pizza). According to an initial study conducted among students, the validation showed a single factor structure and adequate reliability and validity [27]. In 2014, the short and modified version of YFAS was developed that reduced the burden on the participants as it has fewer items than the original version (13 items). The modified YFAS (mYFAS 2.0) version was shown to have psychometric properties similar to YFAS [28].

The mYFAS has been validated in multiple languages, including but not limited to Brazilian [29], Spanish [30], Italian [31, 32], Czech [33], French [34], Chinese [35], and Arabic in Egypt [36]. The German, Turkish, French, Italy, and Czech studies that assessed the validation of the Modified Yale Food Addiction Scale, showed a one-factor structure [37–41], except for the Chinese version (among college students) with a two-factor solution “behavioural symptoms of food addiction” and “adverse consequences of food addiction” [42]. The latter validations provided empirical support for the use of the mYFAS as a reliable and valid tool to assess food addiction.

The Egyptian study [43] revealed that the translated and adapted Arabic version of the YFAS 2.0 is a reliable tool, valid for use in the investigation of food addiction among Arabic-speaking populations. During the translation process, a cross-cultural adaptation was done in the Egyptian study with emphasis on the similarity of meaning rather than the similarity of linguistic form [43]. However, the generalizability of the results is limited by recruiting participants by convenience sampling from a single medical college; adding to the fact that these students may not be representative of the monolingual Arabic-speaking populations for whom the translated scale version is intended [43]. At the end, the authors of the Egyptian study recommended that future research determine whether the one-factor solution obtained in the original mYFAS and other translated versions, could be replicated for the Arabic version [43].

While the Arabic version of the mFAS 2.0 developed and assessed in Egypt is an important addition to the field, there are some limitations, which prohibit its use in other Arabic-speaking countries such as Lebanon. The translation of the questionnaire to a different language might result in non-equivalent measures especially in science/health since the language and culture might influence those outcomes [44, 45] (the language used might differ between the culture in Egypt and that in Lebanon). Regarding the eating pattern and food quality, the traditional Lebanese diet acquired its healthy Mediterranean characteristic from its diversity of food, which appears to have provided healthy ingredients [46]. Though it seems that the Lebanese Mediterranean diet is converging with a pattern high in saturated fat, sugar, and refined foods and is low in fiber, increasing therefore the risk of non-communicable diseases such as obesity, cardiovascular disease, diabetes, and hypertension [47]. On the other hand, in Egypt, the high calorie, carbohydrate, fat, low ash and high trans-fat content in most of the fast foods may be a risk factor for the increase in non-communicable chronic diseases [48].

Therefore, our objective was to assess the psychometric properties of the Arabic version of the mYFAS (mYFAS-Ar-Leb) in terms of number of factors and internal consistency, as well as its validity in the general population in Lebanon. We expect that the mYFAS-Ar-Leb will show one factor (H1) and will have a good internal consistency (H2). We also hypothesize that the food addiction score will positively correlate with mental health issues (depression, anxiety and stress), in line with previous studies [17, 49] (H3).

## Methods

### Participants and procedure

The current study was part of a large cross-cultural project (conducted in Lebanon and Poland) focusing on the Multidimensional Approach to Eating and Obesity (the MATEO study) [50]. In this paper, we will be focusing on the data from Lebanon only. Data was collected between September and November 2020 during the COVID-19 pandemic, when quarantine and social distancing procedures were implemented by the Lebanese Government. The sample was recruited through a snowball technique, from all Lebanese governorates (Beirut, Bekaa, Mount Lebanon, South Lebanon and North Lebanon). The research team contacted people they know, who were asked to forward the link to their contact list via social media applications. Before obtaining the informed consent, the study subjects were notified about the objective of the study and assured of the anonymity of the response. Participants had the right to enroll in this study without any obligation or pressure from the research team, with no monetary compensation given to them for participation.

A total of 1268 persons participated in the present study by filling in an online questionnaire using Google forms. The mean age of the participants was 26.18 years (SD = 11.17; min: 18; max: 85), with 65.1% females and a mean BMI of 24.99 kg/m^2^ (SD = 5.81). Other characteristics are summarized in Table 1.

**Table 1:** Sociodemographic characteristics and weight status of the participants (N = 1268)

Variable	N (%)
Sex	
Male	443 (34.9%)
Female	825 (65.1%)
Marital status	
Single	953 (75.2%)
Married	315 (24.8%)
Education level	
Secondary or less	262 (20.7%)
University	1006 (79.3%)
Weight status (Body Mass Index categories)	
Underweight (< 18.5 kg/m^2^)	76 (6.0%)
Normal weight (18.5–24.99 kg/m^2^)	681 (53.7%)
Overweight (25–29.99 kg/m^2^)	299 (23.6%)
Obesity (≥ 30 kg/m^2^)	212 (16.7%)

### Minimal sample size calculation

A minimal sample of 180 participants was deemed necessary to validate the mYFAS scale, based on 20 participants per 1 scale item [51].

### Measures

Individuals' sociodemographic characteristics included: age, sex, level of education and marital status. Participants self-reported anthropometric measurement (height and weight) in order to compute their body mass index.

The Arabic version of the modified version of the Yale Food Addiction Scale (mYFAS) [28] was utilized in the present study. The scale was first translated from English to Arabic by one psychologist, then back to English from Arabic by another psychologist. The Arabic version was verified by a linguistic professional. The principal investigator compared both English versions to discern any discrepancies; all procedures were done according to the international recommendations of forward-back translation [52]. The mYFAS is composed of 9 core questions including 1 item from each of the symptom groups that compose the 7 diagnostic criteria. The remaining 4 items from the original scale were removed in this version of the YFAS. If a person has at least 3 of the 7 dependence symptoms and meets the criterion for clinical significance, she/he meets food addiction status [16, 28]. In the present study, the Cronbach’s alpha of the mYFAS was 0.859.

The Arabic version [53] of the Depression Anxiety and Stress Scale (DASS-21) was used for the assessment of mental health issues. It is composed of 21 items, scored on a 4-point Likert scale and yields 3 scores for depression, anxiety and stress. The higher the scores, the more the presence of the mental health issue. In this study, the Cronbach’s alpha values were very good for depression (0.850), anxiety (0.845) and stress (0.917). We chose these three variables to assess divergent validity since FA has been previously shown to be associated with mental health issues [54–56].

### Statistical analyses

There was no missing data since the questionnaire was performed and thus all questions were required to continue the online form. A confirmatory factor analysis was carried out using the MPlus v.7.2 software based on polychoric correlation matrix using Weighted Least Squares with Means and Variance Adjusted estimation (WLSMV) method. We also reported several goodness-of-fit indicators: the Relative Chi-square (χ2/df), the Root Mean Square Error of Approximation (RMSEA), the Standardized Root Mean Square Residual, the Comparative Fit Index (CFI) and the Tucker Lewis Index (TLI). The value of χ2 divided by the degrees of freedom (χ2/df) has a low sensitivity to sample size and may be used as an index of goodness of fit (cut-off values: < 2–5). Values of CFI and TLI ≥ 0.90, RMSEA ≤ 0.08 [57] and SRMR < 0.05 [58] indicate a good fit of the model.

Data analysis was conducted using SPSS software version 23. Cronbach’s alpha was recorded for reliability analysis of all scales. The normality of distribution of age, food addiction, stress, depression and anxiety scores was confirmed via a calculation of the skewness and kurtosis; values for asymmetry and kurtosis between − 2 and + 2 are considered acceptable in order to prove normal univariate distribution [59, 60], in samples larger than 300 [61]. The Chi-square test was used to compare two categorical variables, whereas the Student t test was used to compare two means. Pearson correlation test was used to correlate two continuous variables; in psychological research, values of 0.1 were considered small correlations, whereas values of 0.2 and > 0.3 were classified as being moderate and large correlations respectively [62, 63]. *p* < 0.05 was considered statistically significant.

## Results

### Factor validity

**Fig. 1:**
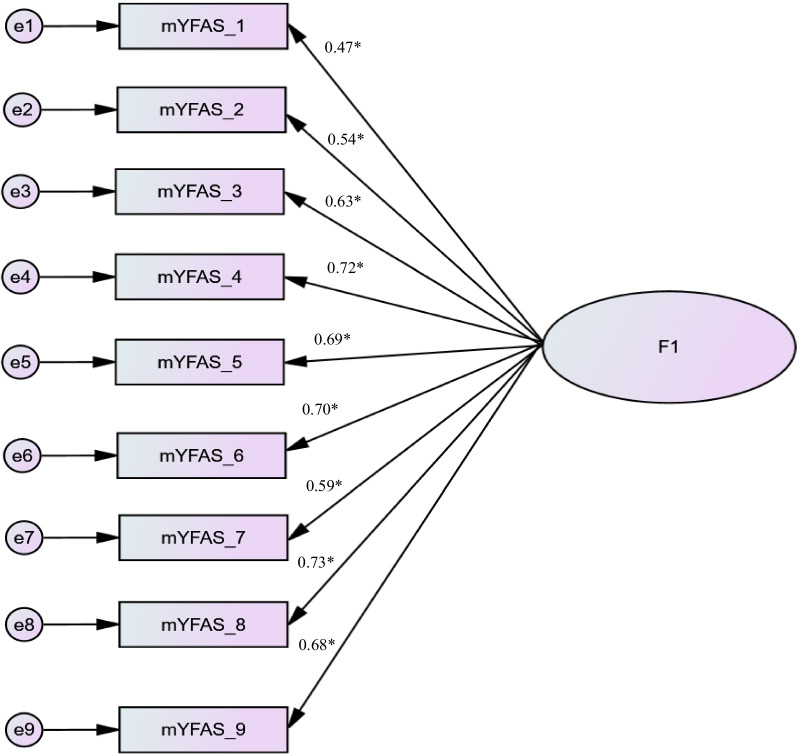
Standardized factor loadings of the one-factor model of the Arabic version of the modified Yale Food Addiction Scale (mYFAS-Ar-Leb) in Lebanese general population. *p < 0.001

A confirmatory factor analysis was performed on the one-factor structure among the total sample (N = 1268). The following results were obtained: χ2/df = 82.15/27 = 3.04, RMSEA = 0.04 [0.03–0.05], SRMR = 0.026, CFI = 0.98 and TLI = 0.97. The standardized factor loadings are summarized in Fig. 1.

### Internal consistency

The Cronbach’s alpha value was good for the total scale (0.859). The total-item correlations varied between 0.583 and 0.744 (Table 2).

**Table 2:** Correlation between the total score and each item of the scale

Item	Total-item correlation
mYFAS 1	0.583
mYFAS 2	0.624
mYFAS 3	0.694
mYFAS 4	0.733
mYFAS 5	0.710
mYFAS 6	0.741
mYFAS 7	0.653
mYFAS 8	0.744
mYFAS 9	0.710

Numbers refer to Pearson correlation coefficients; *p* < 0.001 for all correlations

### Divergent validity

The mYFAS-Ar-Leb score was positively and strongly associated with the DASS-21 score related to stress (r = 0.582; *p* < 0.001), anxiety (r = 0.542; *p* < 0.001) and depression (r = 0.532; *p* < 0.001).

### Prevalence of food addiction and association with age, sex and BMI

The percentage of participants with food addiction was 226 (17.8%) in the total sample, with a significantly higher percentage of food addiction found among participants with overweight and obesity (n = 130) compared to those who had a normal weight (n = 96) (25.4% vs 12.7%; *p* < 0.001).

No difference in terms of food addiction was found between men and women (19.6% vs 16.8%; *p* = 0.216) and between those with a university level of education vs secondary or less (16.9% vs 21.4%; *p* = 0.092), whereas a significantly higher mean age was found in those with food addiction compared to those without food addiction (29.26 ± 13.94 vs 25.51 ± 10.37; *p* < 0.001; Cohen’s d = 0.305).

## Discussion

The one-factor structure that has been previously found in multiple validation papers (German [37], Turkish [40], French [39], Czech [41], and Italian [38]), was confirmed in our sample. In terms of reliability, the Cronbach’s alpha value obtained in the present study (0.859) was higher than the one found in the Turkish (0.70) [40], German (0.82) [37], and French (0.79) [39] versions, but a little lower than the one found in the Czech version (0.89) [41]. In addition, mYAS-AR-Leb score was significantly and strongly associated with higher mental health issues in the present study; these findings corroborate the ones found in a meta-analysis [17], which showed that there is a connection between some criteria of mental illness and those of food addiction. The correlations between mYFAS-Ar-Leb and DASS-21 scores were large (r > 0.5 for all variables); Correlation coefficients below 0.707 indicate that less than half of the variance of FA is explained by mental health issues. In fact, this high correlation between FA and mental health issues in our study corroborate previous findings [23, 64, 65] and suggest a kind of overlap between the two entities. A person’s appraisal of his body involves cognitive, behavioral, and emotional aspects; consequently, a positive relationship exists between food addiction and mental health symptoms [17]. Subsequently, our results show that the Arabic version of the mYFAS is a valid tool to screen for the presence of food addiction among the Lebanese population.

The prevalence of food addiction in our sample was 17.8%, a similar percentage to the one found in a meta-analysis (16.2%) [17], but higher than the ones found in Turkey (11.8%) [40], USA (11.4%) [27], Germany (8.8%) [66], and France (8.7%) [67] (using the YFAS scale in those four countries). Multiple factors were previously shown to be related to more food addiction, including but not limited to emotions and stress [68], brain chemistry [69], trauma [70] and genetics [20]. This high prevalence in terms of food addiction among Lebanese may be explained by the fact that Lebanese dishes are rich in salt but low in sugar [71]. Another study showed that Lebanese consume traditional dishes rich in saturated fat, which puts the health of the population in danger in terms of coronary disease [72]. The prevalence of food addiction obtained in this study might have been affected by the COVID-19 pandemic; it has been previously documented that the number of meals consumed per day, intake of sweets, salty snacks, and caffeinated and sweetened beverages increased during the pandemic among the Lebanese population [73].

A study based on neuroimaging on animals and humans showed that foods rich in sugar, fat, and/or salt, have an effect on the central nervous system similar to that exerted by addictive drugs [74]. It is important to state that the mYFAS 2.0 scale has a lower symptom count and diagnostic threshold score, compared to the full YFAS 2.0. This was kind of expected since the full original scale produces an increased count of symptoms compared to the shorter version since the responder can acknowledge the presence of one symptom in different questions, whereas the shorter version (mYFAS 2.0) allows only one question per symptom [75]. Subsequently, the full scale showed a marginally higher FA prevalence as assessed by the ‘diagnostic’ threshold compared to its shorter version, but the clinical importance of this difference remains unknown. As a result, the mYFAS 2.0 may be suitable in studies that seek specificity over sensitivity, but the full version of this scale remains favored when a more sensitive tool of addictive-like eating behavior is needed [75].

Participants with food addiction had a higher Body Mass Index; while some studies have showed a strong relationship between those two variables (similar to our results) [41, 76, 77], Meule et al. [78] found a weak correlation between these two variables. This might be due to the fact that food consumption has a rewarding effect on the neural system, which possibly contributes to increased overweight and obesity worldwide [79, 80]. The controversy can be explained by the fact that obesity is a multifaceted condition in which there is no single cause or solution exists and addiction is marked by lack of control over consumption without clear boundaries of food addiction like substance abuse [81]. Despite that, further studies are needed to understand the relation between food addiction and Body Mass Index and the reasons behind it.

A significantly higher mean age was found in participants with higher YFAS scores compared to lower YFAS scores. Previous findings showed that higher food addiction was found in younger participants (18–29 years of age) [41, 82]. Our sample had a low mean age (26.18 years), therefore, our results should be interpreted with caution. Young adults in Lebanon might be attracted by the consumption of out-of-home meals [83] and be more influenced by food rich in calories [84].

The study findings did not show any difference in food addiction between males and females, in agreement with the results of other studies [82, 84, 85]. However, other studies have demonstrated a higher prevalence of FA in women, which can be interpreted by the cultural differences between countries in terms of eating habits and food addiction [18, 86].

Research explaining the association between education and FA is scarce; one study showed the impact of nutrition education on food addiction symptoms and minor enhancements in FA markers in university students [87]. Our study did not show an association between education and FA. The results can be explained by the fact that there is an unequal distribution in our sample in terms of education (the majority of the participants had a university level). Thus, future studies involving a more homogenous distribution of the sample are needed.

The prevalence of obesity has been increasing worldwide, with the existing weight loss techniques showing to be widely ineffective. Consequently, researchers are now considering the possible addictive aspects of food on reward and control paths in the brain and its related behavior (just like drugs), with treatments for addictive disorders showing efficacy in FA as well [88]. One of the greatest potential advantages of identifying the similarities between substance addictions and overeating is the development of effective interventions. The usual tactic used (dieting and physical exercise) was shown to have low adherence, which might be due to treatment of the outcome rather than the cause behind it. Consequently, a model about FA may help recognize the basics of overweight/obesity further than the absence of willpower, which can be more beneficial for the implementations of interventions and policies [89, 90]. Thus, the current study plays an integral role in highlighting the FA prevalence in Lebanon, which might be a first step towards raising awareness about minimizing the craving for the intake of addictive food and stressing on the importance of early treatment plans aiming at preventing addiction occurrence and halting addiction progression.

### Limitations

Due to the cross-sectional study design no causal relationships should be inferred. A selection bias is possible since participants had a low mean age, a university level of education; plus, women outnumbered men. Self-reporting symptoms without the evaluation of a healthcare professional, and self-reporting anthropometric information (weight and height) predisposes us to an information bias. The collection of data online during the COVID-19 pandemic period may have affected the reliability of the study. This may have affected psychological distress and food addiction as well. We did not assess the presence of binge eating in this sample, which could have impacted the results of our study since there is a strong overlap between symptoms of food addiction and binge eating [13, 14]. The sample was collected via a snowball technique, which hinders the generalizability of the results. More psychometric properties are needed in future studies (test–retest, convergent validity with other scales to assess eating attitudes).

## Conclusion

Overall, the mYFAS 2.0 is psychometrically similar to the full YFAS 2.0 and an appropriate alternative and briefer screening tool compared to the full YFAS 2.0. Researchers and clinicians in Lebanon can use the Arabic version of the mYFAS in their research and clinical practice. The initial psychometric properties of this scale are encouraging, with further studies needed to validate this scale among other Arabic-speaking countries. Our study findings also highlighted that the use of the mYFAS 2.0 in Lebanese population might help estimate FA prevalence and stress on the need for effective treatment and preventive measures to craving for addictive foods.

## Declarations

### Availability of data and materials

All data generated or analyzed during this study are not publicly available to maintain the privacy of the individuals’ identities. The dataset supporting the conclusions is available upon request to the corresponding author.
